# Overexpression of artificially fused bifunctional enzyme 4CL1–CCR: a method for production of secreted 4-hydroxycinnamaldehydes in *Escherichia coli*

**DOI:** 10.1186/s12934-015-0309-2

**Published:** 2015-08-12

**Authors:** Shuxin Liu, Qi Qi, Nan Chao, Jiayin Hou, Guodong Rao, Jin Xie, Hai Lu, Xiangning Jiang, Ying Gai

**Affiliations:** College of Biological Science and Technology, Beijing Forestry University, Beijing, 100083 People’s Republic of China; The Tree and Ornamental Plant Breeding and Biotechnology Laboratory of Chinese Forestry Administration, National Engineering Laboratory for Tree Breeding, Beijing, 100083 People’s Republic of China; Research Institute of Forestry, Chinese Academy of Forestry, Beijing, 100091 People’s Republic of China; Institute of Agricultural Bio-Resources, Hunan Academy of Agricultural Sciences, Changsha, 410125 People’s Republic of China

**Keywords:** Bifunctional enzyme, 4-Coumaric acid: coenzyme A ligase, Cinnamoyl coenzyme A reductase, Biotransformation, 4-Hydroxycinnamaldehydes, *Escherichia coli*

## Abstract

**Background:**

4-Hydroxycinnamaldehydes are important intermediates in several secondary metabolism pathways, including those involved in the biosynthesis of phenolic acids, flavonoids, terpenoids and monolignols. They are also involved in the biosynthesis and degradation of lignins, which are important limiting factors during the processes of papermaking and biofuel production. Access to these aromatic polymers is necessary to explore the secondary biometabolic pathways they are involved in. Coniferaldehyde, sinapaldehyde, *p*-coumaraldehyde and caffealdehyde are members of the 4-hydroxycinnamaldehyde family. Although coniferaldehyde and sinapaldehyde can be purchased from commercial sources, *p*-coumaraldehyde and caffealdehyde are not commercially available. Therefore, there is increasing interest in producing 4-hydroxycinnamaldehydes. Here, we attempted to produce 4-hydroxycinnamaldehydes using engineered *Escherichia coli*.

**Results:**

4-Coumaric acid: coenzyme A ligase (4CL1) and cinnamoyl coenzyme A reductase (CCR) were fused by means of genetic engineering to generate an artificial bifunctional enzyme, 4CL1–CCR, which was overexpressed in cultured *E. coli* supplemented with phenylpropanoic acids. Three 4-hydroxycinnamaldehydes, *p*-coumaraldehyde, caffealdehyde and coniferaldehyde, were thereby biosynthesized and secreted into the culture medium. The products were extracted and purified from the culture medium, and identically characterized by the HPLC–PDA–ESI–MSn. The productivity of this new metabolic system were 49 mg/L for *p*-coumaraldehyde, 19 mg/L for caffealdehyde and 35 mg/L for coniferaldehyde. Extracellular hydroxycinnamoyl-coenzyme A thioesters were not detected, indicating that these thioesters could not pass freely through the cellular membrane. The fusion enzyme 4CL1–CCR can catalyze sequential multistep reactions, thereby avoiding the permeability problem of intermediates, which reveals its superiority over a mixture of individual native enzymes. Moreover, we have described a highly sensitive and selective method for separation and identification of phenylpropanoic acids and their corresponding cinnamaldehydes in the present paper. The feasibility of this method has been proven in the application of the method to the analysis of the metabolites of whole-cell catalysts.

**Conclusions:**

We have established a bioconversion pathway for the microbial production of valuable 4-hydroxycinnamaldehydes from phenylpropanoic acids. This biotransformation method is both convenient and environmentally friendly, and provides new insights into the biosynthesis of natural plant secondary products.

## Background

4-Hydroxycinnamaldehydes are a class of natural plant secondary products that includes coniferaldehyde, sinapaldehyde, *p*-coumaraldehyde and caffealdehyde. They are involved in several secondary metabolism pathways, such as those involved in the biosynthesis of phenolic acids, monolignols, flavonoids and terpenoids. 4-Hydroxycinnamaldehydes are also key intermediates in the biosynthesis and degradation of lignins, which protect cell wall polysaccharides from microbial degradation [1]. These aromatic polymers are not only responsible for the characteristic phloroglucinol staining of lignified tissues, but also for much of the recalcitrance in converting plant biomass to pulp or liquid fuels [2]. In addition, 4-hydroxycinnamaldehydes are also involved in many biosynthetic pathways of plant natural products, including secondary metabolites with regulatory or biomedical functions [3, 4]. It was reported that 4-hydroxycinnamaldehyde from *Alpinia galanga* can induce human leukemic cell apoptosis through a combination of mitochondrial and endoplasmic reticulum stress pathways [4].

Further study of these cinnamaldehydes would enable us to better understand how lignin is biosynthesized, which will have a significant impact on plant biotechnology. So far, only coniferaldehyde and sinapaldehyde can be purchased from commercial sources, while *p*-coumaraldehyde and caffealdehyde are not commercially available. The extraction method of aldehydes from plants has some limitations, such as the long growth time, low yield, and environmental consequences of harvesting considerable plant biomass. However, the high complexity of plants also introduces additional challenges. Exploration of other downstream enzymes in the lignin metabolic pathway has been hampered by limited capability to extract cinnamaldehydes. Driven by the need for 4-hydroxycinnamaldehydes, a series of chemical synthetic methods have been developed, including oxidation of 4-hydroxylcinnamyl alcohols, reduction of phenylpropanoic acids, etc. [5–9]. However, these chemical strategies present several obstacles, such as complicated multiple steps, excessive by-products and difficulty of purification. One alternative to chemical synthesis is to produce these aldehydes in microorganisms.

Whole-cell biotransformation refers to the use of live organisms (often microbes) to carry out a chemical reaction that is more costly or not feasible to perform by non-biological means. Throughout years of development, biotransformation models have moved from a theoretical stage into successful experimental verification [10, 11]. A new trend in the production of useful phytochemicals is to use biotransformation [12]. The essence of biotransformation is that enzymes from organisms are exploited to carry out chemical reactions [13]. *Escherichia coli* (*E. coli*) is a commonly used system for producing plant natural products [14]. Compared with other microbes, *E. coli* has a much shorter growth time, a more clear genetic background and is more amenable to genetic manipulation. Several important plant metabolites have been successfully produced in *E. coli*, including flavonoids, terpenoids and hydroxycinnamates [15–19]. By constructing a functional pathway in *E. coli*, the target compound can be produced and engineered for increased yield.

**Fig. 1:**
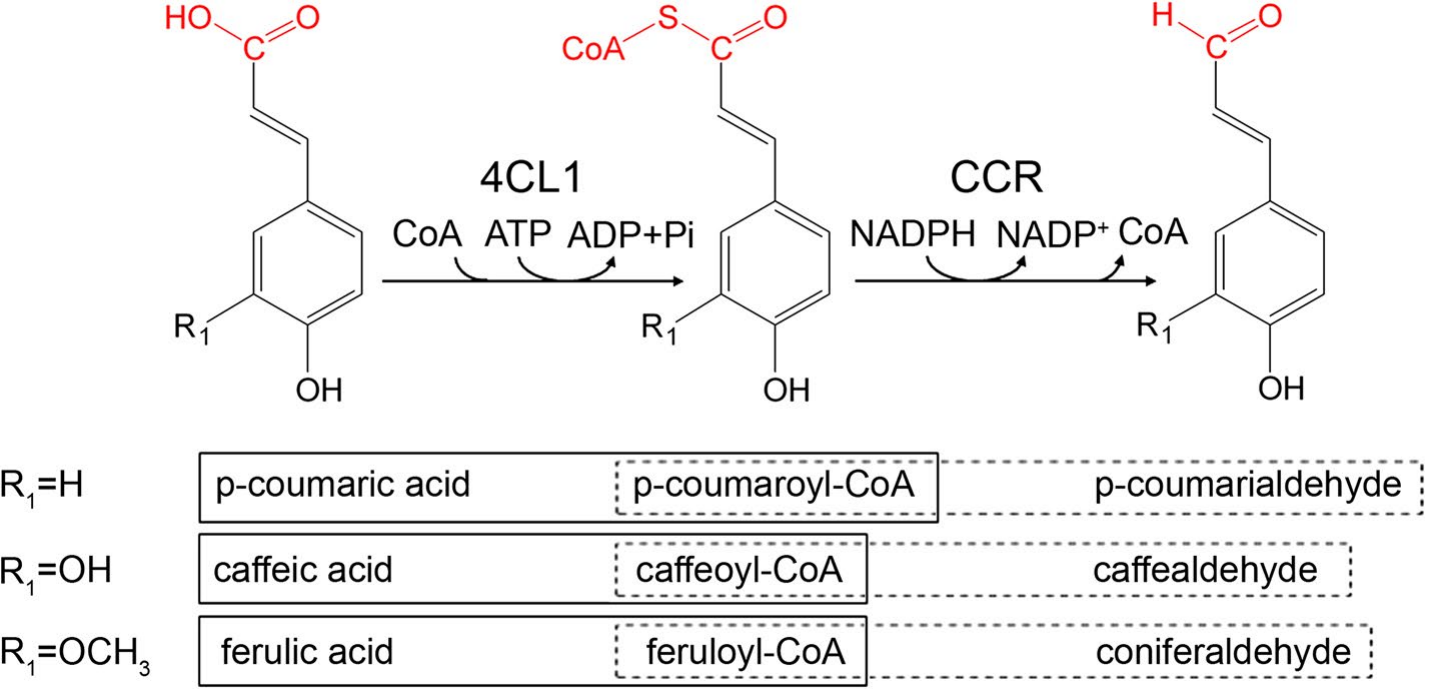
The 4-hydrocinnaldehydes biosynthesis reactions catalyzed by 4CL1 and CCR. The prevalent conversions occurring in *P. tomentosa* are outlined (*solid boxes* for 4CL1 and *dashed boxes* for CCR).

The formation of 4-hydroxycinnamaldehydes is catalyzed by 4-coumaric acid: coenzyme A ligase (4CL1) and cinnamoyl coenzyme A reductase (CCR). 4CL1 from *Populus tomentosa* (*P. tomentosa*) catalyzes the reaction of phenylpropanoic acids to their corresponding hydroxyphenylacetyl-CoA thioesters in the presence of adenosine triphosphate (ATP) and coenzyme A (CoA) [20]. CCR subsequently converts hydroxycinnamoyl-CoA thioesters to their corresponding cinnamaldehydes in the presence of NADPH (Fig. 1) [21]. A fusion gene encoding 4CL1–CCR was designed and its expression vector was constructed. The recombinant protein 4CL1–CCR can be efficiently expressed in *E. coli*. The fusion enzyme 4CL1–CCR has a relatively high catalytic efficiency as both 4CL1 and CCR, which confers the ability to facilitate the reaction from phenylpropanoic acids to 4-hydroxycinnamaldehydes. The intermediate product 4-hydroxycinnamoyl-CoA is unstable and hard to obtain. With the help of this fusion enzyme we were able to realize the sequential reactions, eliminating the necessity to synthesize the intermediate product, which is more convenient and cost saving.

Here, we attempted to produce 4-hydroxycinnamaldehydes using engineered *E. coli* as a whole-cell biocatalyst. When the engineered *E. coli* cells were cultured in media supplemented with *p*-coumaric acid, caffeic acid and ferulic acid respectively, a high yield of *p*-coumaraldehyde, caffealdehyde and coniferaldehyde were obtained. The production of 4-hydroxycinnamaldehydes by the biotransformation method has the advantages of being low in production cost as well as a convenient procedure, which provides new insights for future cost-effective commercial development. Although the modification described here is merely a proof-of-concept that provides a foundation for further developments in *E. coli* engineering, it is likely that it could be used for production of other natural products.

## Results

### SDS-PAGE analysis of recombinant enzymes

**Fig. 2:**
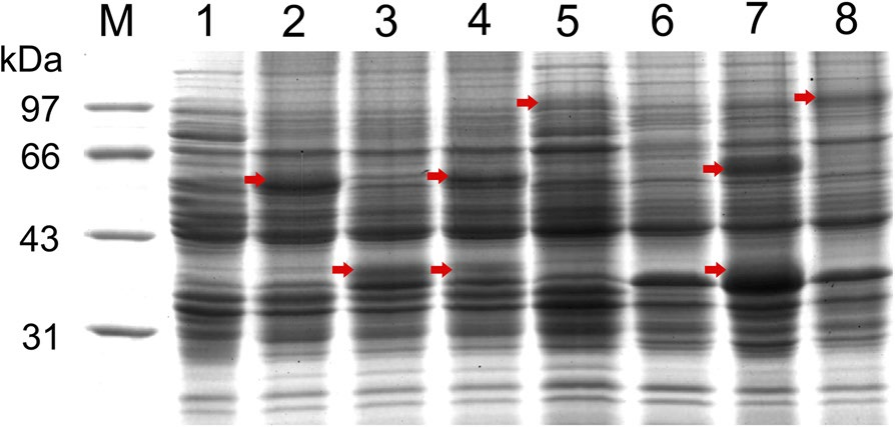
SDS-PAGE analysis of recombinant enzymes. *Lane M* protein molecular markers. *Lanes 1*–*5*, total crude proteins from the induced cells of *E. coli* strains M15: wild type, M-4CL1, M-CCR, M-4CL1+M-CCR, M-4CL1–CCR, respectively; *Lanes 6–8*, total crude proteins from the induced cells of *E. coli* strains BL21: wild type, B-4CL1+CCR, B-4CL1–CCR, respectively. The *red arrows* point to the target proteins. The *numbers* on the *left* indicate the molecular weight (MW) of the markers (in kilodaltons, kDa).

A gel electrophoretogram of total crude proteins extracted from different strains is shown in Fig. 2. The wild type *E. coli* strains M15 and BL21 were used as the negative control (Fig. 2 lanes 1 and 6). Compared with lane 1, the intensity of the protein band around 60 kDa in lane 2 was significantly increased, which corresponded with the expression of 4CL1. Similarly, the target proteins expressed in *E. coli* strains M-CCR, M-4CL1+M-CCR, M-4CL1–CCR, B-4CL1+CCR, B-4CL1–CCR were observed at the specific location (Fig. 2 lanes 3, 4, 5, 7 and 8). As expected, the molecular masses of 4CL1, CCR, and 4CL1–CCR were observed to be approximately 60, 40 and 100 kDa, respectively. The SDS-PAGE profiles revealed that target proteins from *P. tomentosa* were efficiently expressed in *E. coli*. Thus, the strains mentioned in this study can be applied to follow-up experiments focusing on whole-cell biocatalysts.

### Production of 4-hydroxycinnamaldehydes in *E. coli*

**Fig. 3:**
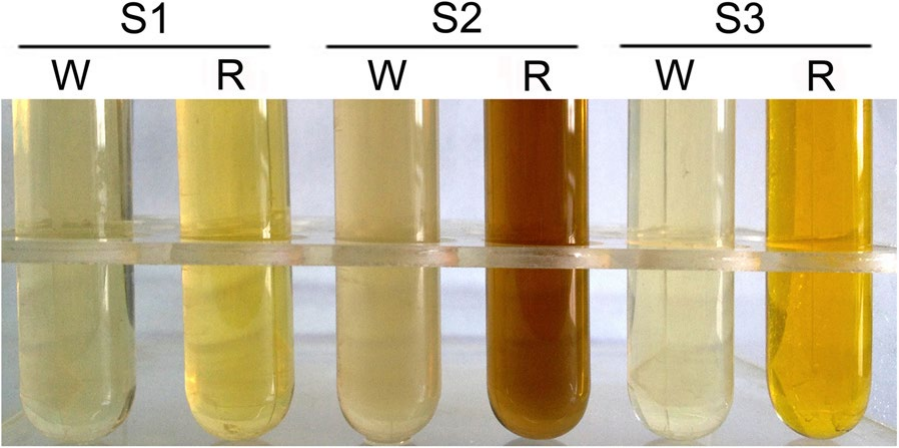
Color change of the medium due to fusion enzyme 4CL1–CCR. W, wild type *E. coli* strains M15; R, recombinant *E. coli* strains M-4CL1–CCR; *S1* *p*-coumaric acid; *S2* caffeic acid; *S3* ferulic acid.

A series of whole-cell conversion studies were carried out to verify the feasibility of using 4CL1–CCR, an exogenous bifunctional enzyme, to produce 4-hydroxycinnamaldehydes. Interestingly, the color of the culture medium supplemented with three kinds of phenylpropanoic acids did not change in culture containing the wild type *E. coli* strains M15, but turned pale yellow, brown and brilliant yellow, respectively, in the culture containing the recombinant strains M-4CL1–CCR (Fig. 3). These color variations might be caused by the formation of new compounds. Further verification and analysis by HPLC–PDA–ESI–MSn are presented in subsequent sections.

**Fig. 4:**
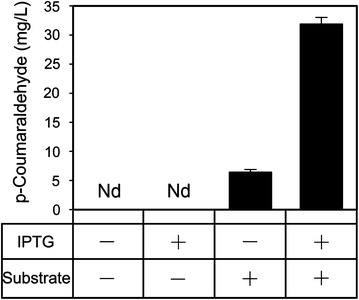
In vivo enzyme activity of 4CL1–CCR towards substrate *p*-coumaric acid. The concentration of *p*-coumaraldehyde was measured in each culture medium with (+) or without (−) the addition of IPTG and substrate. *Error bars* indicate mean values ± SD from three independent experiments. *Nd* not detected.

To verify that the newly introduced 4-hydroxycinnamaldehyde synthesis pathway was functional and that the increased 4-hydroxycinnamaldehyde production resulted from action by bifunctional enzyme 4CL1–CCR, the *p*-coumaraldehyde concentrations were determined under various culture conditions. As shown in Fig. 4, the new compound was detected in the medium of *E. coli* M-4CL1–CCR with externally added *p*-coumaric acid as a substrate. Meanwhile, no *p*-coumaraldehyde was detected in the medium without exogenous *p*-coumaric acid, either in the presence or absence of IPTG. This phenomenon indicates that the microorganism itself does not have the capability to synthesize 4-hydroxycinnamaldehydes. The bifunctional enzyme 4CL1–CCR plays a central role in cellular metabolism by converting phenylpropanoic acids to their corresponding cinnamaldehydes. After induction by IPTG, the *p*-coumaraldehyde in the M-4CL1–CCR was increased by more than fourfold, compared with not adding IPTG.

**Fig. 5:**
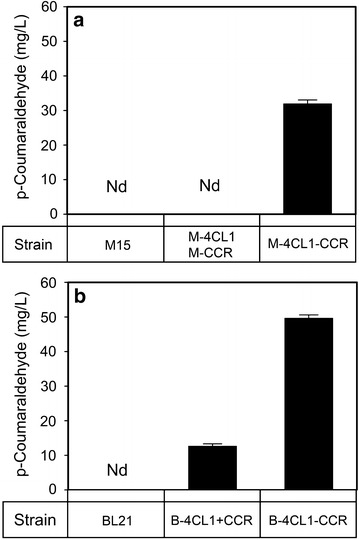
In vivo production of *p*-coumaraldehyde in different *E. coli* strains. **a** production of *p*-coumaraldehyde with strains M15, M-4CL1+M-CCR and M-4CL1–CCR; **b** production of *p*-coumaraldehyde with strains BL21, B-4CL1+CCR and B-4CL1–CCR. All strains were cultivated in LB medium with 1 mM *p*-coumaric acid at 37 °C for 16 h. *Error bars* indicate mean values ± SD from three independent experiments. *Nd* not detected.

To compare the in vivo enzymatic activity of the bifunctional enzyme 4CL1–CCR and the mixture of individual native enzymes 4CL1 and CCR, the equivalent amount of *p*-coumaric acid was added into the medium of several *E. coli* strains. Cultures of the wild type *E. coli* strains M15 were grown as a negative control. In the medium of *E. coli* M-4CL1–CCR, a large amount of *p*-coumaraldehyde was detected. However, extracellular free *p*-coumaraldehyde was not detected in the co-culture of the M-4CL1 with the M-CCR (Fig. 5a). To investigate the cause of this phenomenon, a subsequent experiment was carried out to detect *p*-coumaroyl-CoA, which is an intermediate product in the biosynthesis of *p*-coumaraldehyde. Interestingly, analysis of the culture medium of *E. coli* M-4CL1 revealed that *p*-coumaroyl-CoA was not detectable, which demonstrates that *p*-coumaroyl-CoA could not freely pass through the cellular membrane. That is, hydroxycinnamoyl-CoA thioesters accumulate only in the cell. There was no *p*-coumaroyl-CoA present to act as a substrate for CCR; therefore, no reaction product was detected. This finding suggests that use of recombinant strains is not also feasible because of the transmembrane problem. Thus the mixture of individual native enzymes 4CL1 and CCR is less efficient than the bifunctional enzyme in the conversion of *p*-coumaric acid into *p*-coumaraldehyde in our system.

**Fig. 6:**
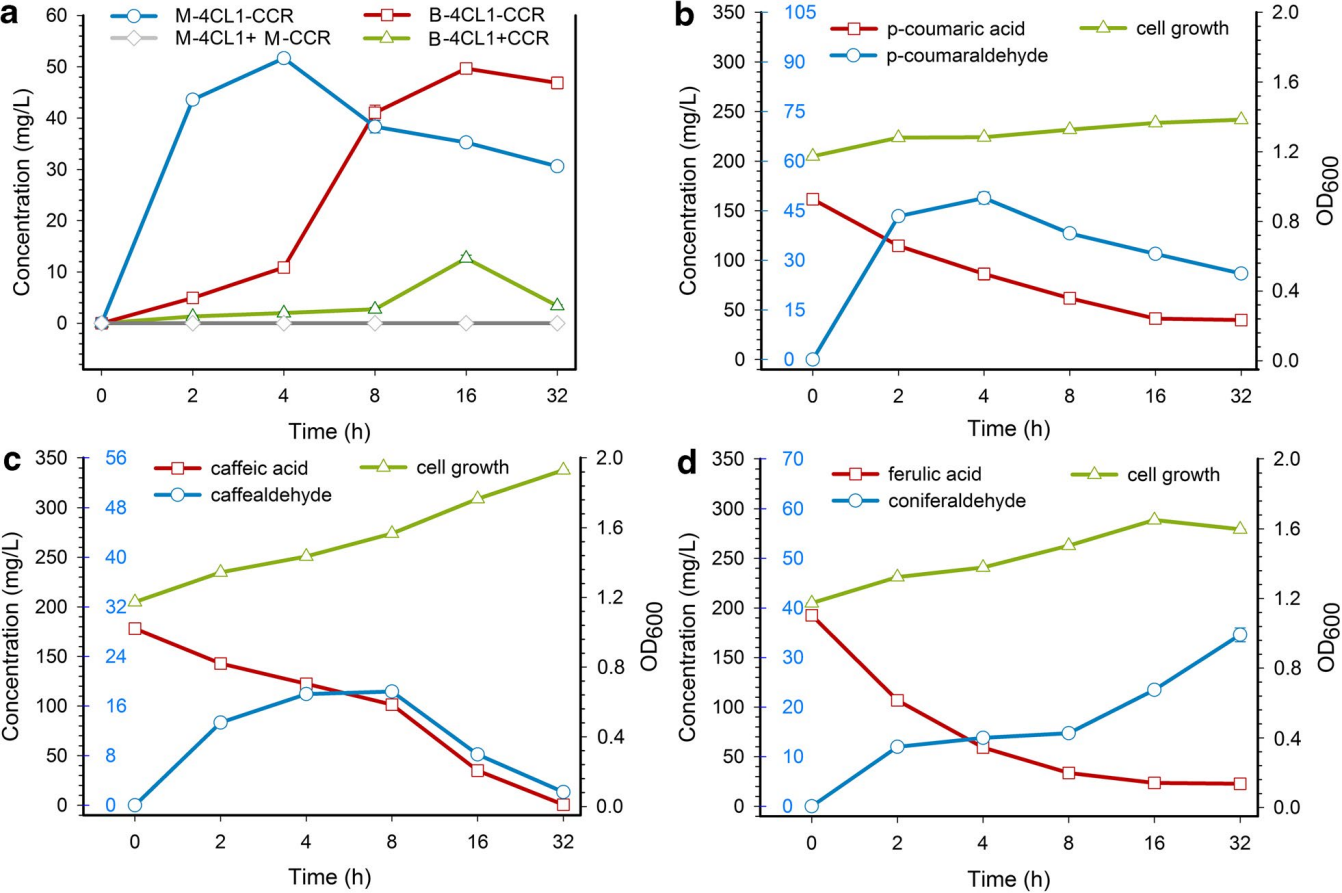
Time-course profiles of 4-hydrocinnaldehydes production. **a** Production of *p*-coumaraldehyde in four different *E. coli* strains: M-4CL1+M-CCR, M-4CL1–CCR, B-4CL1+CCR, B-4CL1–CCR; **b** production of *p*-coumaraldehyde and consumption of *p*-coumaric acid in *E. coli* strains M-4CL1–CCR; **c** production of caffealdehyde production and consumption of caffeic acid in *E. coli* strains M-4CL1–CCR; **d** production of coniferaldehyde and consumption of ferulic acid in *E. coli* strains M-4CL1–CCR. Growth curves of *E.* *coli* are also shown in the graph. *Error bars* indicate mean values ± SD from three independent experiments.

As a control experiment, we also compared the in vivo enzymatic activity of the fusion expression system and the co-expression system. Cultures of the wild type *E. coli* strains BL21 were grown as a negative control. *E. coli* strains B-4CL1+CCR with pE-4CL1+CCR, and B-4CL1–CCR with pE-4CL1–CCR, were tested for production of *p*-coumaraldehyde. The transformant B-4CL1–CCR produced higher concentration of *p*-coumaraldehyde (49 mg/L), whereas the titer of *p*-coumaraldehyde produced by the transformant B-4CL1+CCR was 13 mg/L (Fig. 5b). According to the biotransformation experiment, a significant difference in the maximum amount of *p*-coumaraldehyde was shown between B-4CL1–CCR and B-4CL1+CCR, but little difference between B-4CL1–CCR and M-4CL1–CCR (Fig. 6a). In general, *E. coli* strain M-4CL1–CCR has the advantage of fast reaction rate and high conversion ratio for the biotransformation of *p*-coumaric acid.

### Characterization of phenylpropanoic acids and 4-hydrocinnaldehydes by HPLC–PDA–ESI–MSn

**Fig. 7:**
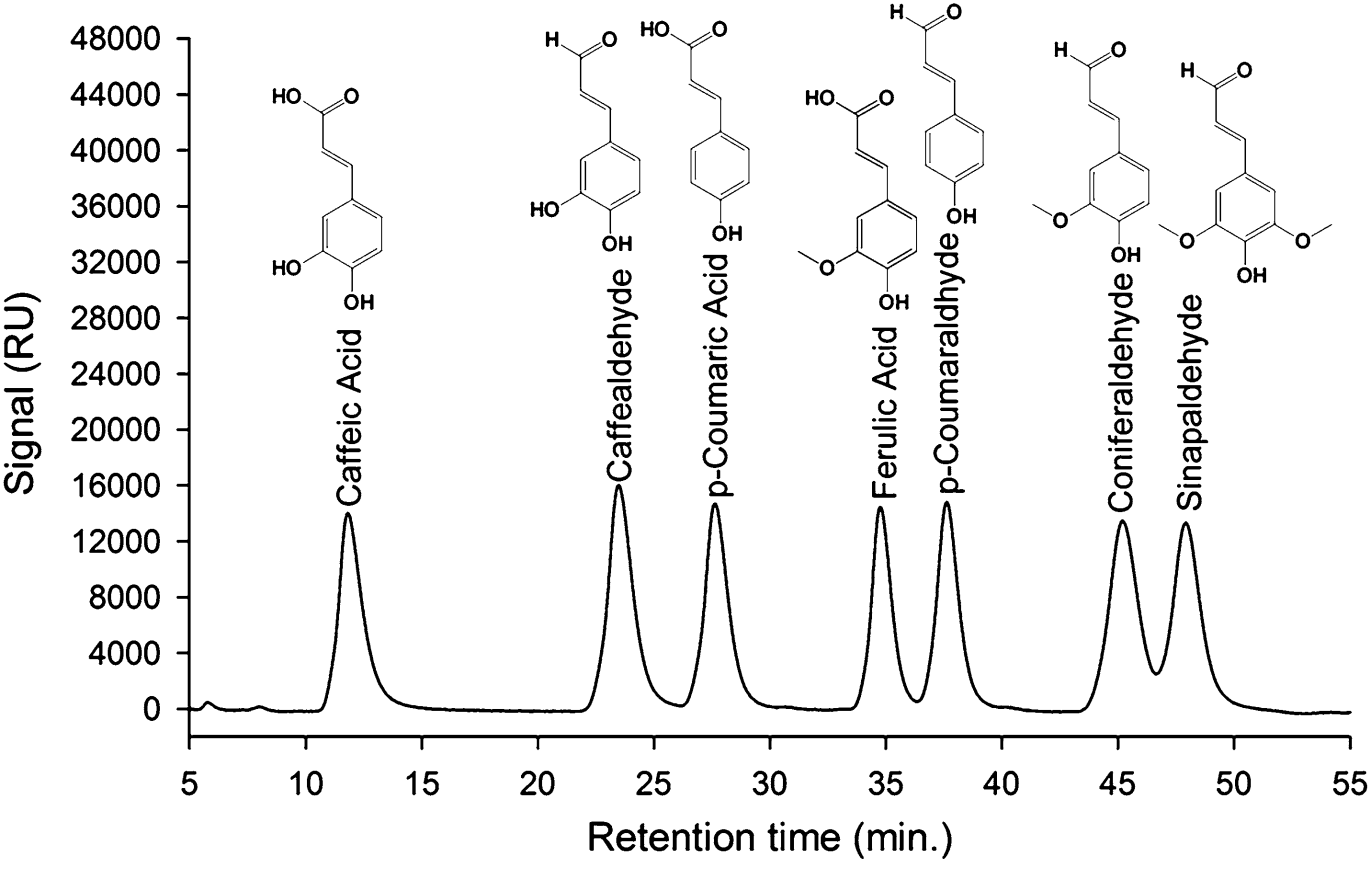
HPLC–PDA separation and ESI-Ion-trap-MS detection of phenylpropanoic acids (substrates) and 4-hydrocinnaldehydes (products). From *left to right* caffeic acid, caffealdehyde, *p*-coumaric acid, ferulic acid, *p*-coumaraldhyde, coniferaldehyde and sinapaldehyde.

For accurate and rapid identification of the metabolites, a highly sensitive and selective HPLC–PDA–ESI–MSn method was established. A solution containing phenylpropanoic acids and 4-hydrocinnaldehydes was separated by high-performance liquid chromatography (HPLC). Seven compounds were completely separated, which indicates that this HPLC separation condition was acceptable for our samples (Fig. 7). The retention times for each compound are shown in Table 1. Note that the chromatography of *p*-coumaraldehyde and caffealdehyde were not obtained from standards but from analyses of our samples. Based on signal intensities and the signal-to-noise ratios, a negative ion scan mode was chosen for phenylpropanoic acids and a positive ion scan mode was chosen for 4-hydrocinnaldehydes. The specific ions of precursors and products of selected reaction monitoring (SRM) of tandem mass spectrometry (MS2) are summarized in Table 2.

**Table 1:** Linear regression of phenylpropanoic acids and 4-hydrocinnaldehydes for quantitative analysis

Compound	Retention time (min)	Regression equation	Linearity range (pmol)	Correlation coefficient (*R* ^2^)
*p*-Coumaric acid	27.16	y = 56,836x – 34,958	40.93–13,641.90	0.9998
Caffeic acid	12.41	y = 107,373x + 2,2054	13.65–4,551.51	0.9998
Ferulic acid	34.28	y = 144,834x – 7,23452	11.43–3,810.90	0.9958
*p*-Coumaraldehyde	37.32	–	–	–
Caffeldehyde	23.42	–	–	–
Coniferaldehyde	44.69	y = 542,502x – 9,79192	3.70–1,234.64	0.9957
Sinapaldehyde	47.53	y = 330,008x – 3,86290	3.75–1,248.74	0.9986

**Table 2:** Optimized MS(n) condition for detection of phenylpropanoic acids and 4-hydrocinnaldehydes

Compound	Scan mode	Precursor ion	Collision energy (eV)	Product ion of SRM mode (MS2)
*p*-Coumaric acid	−	164	35	119
Caffeic acid	−	180	34	135
Ferulic acid	−	194	33	149, 178
*p*-Coumaraldehyde	+	148	33	121, 131
Caffealdehyde	+	164	32	147
Coniferaldehyde	+	178	33	147, 161
Sinapaldehyde	+	208	30	177, 191

### Analysis of the whole-cell catalysis process of phenylpropanoic acids

**Fig. 8:**
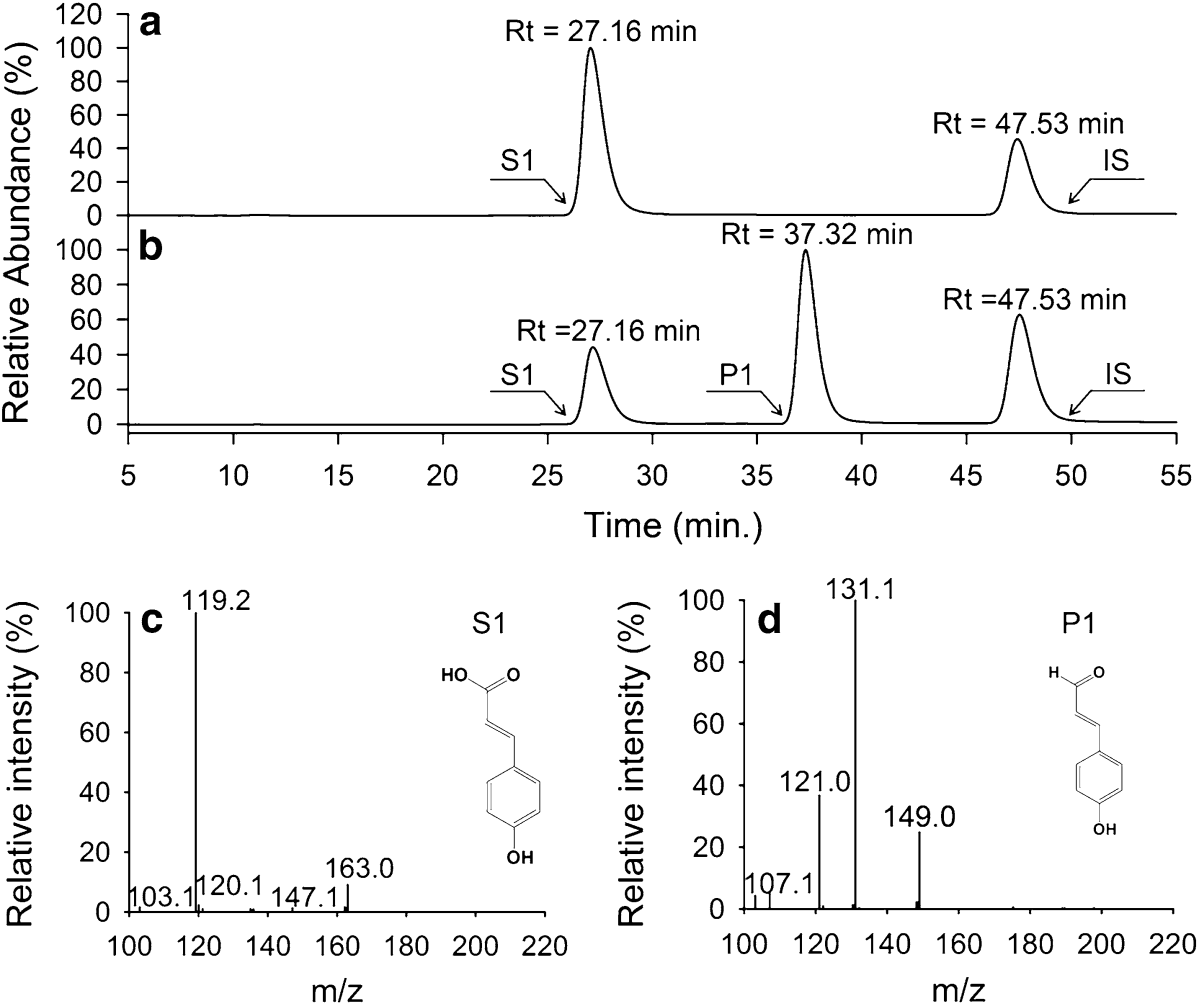
Production of *p*-coumaraldehyde in *E. coli* M-4CL1–CCR. **a** *p*-coumaric acid (S1), Internal standard (IS); **b** reaction product of *p*-coumaric acid (P1); **c** MS/MS profile of S1; **d** MS/MS profile of P1.

**Fig. 9:**
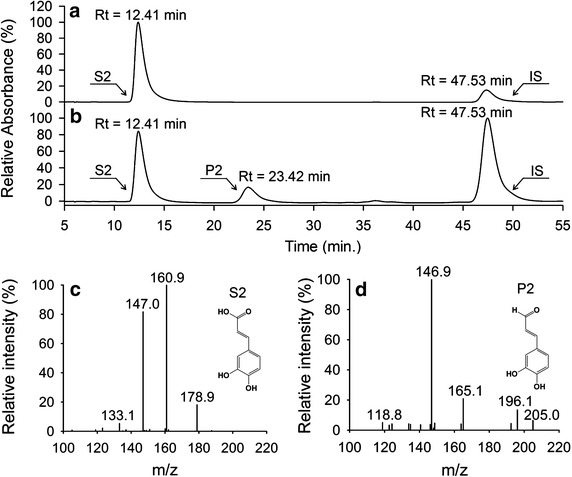
Production of caffealdehyde in *E. coli* M-4CL1–CCR. **a** caffeic acid (S2), Internal standard (IS); **b** reaction product of caffeic acid (P2); **c** MS/MS profile of S2; **d** MS/MS profile of P2.

**Fig. 10:**
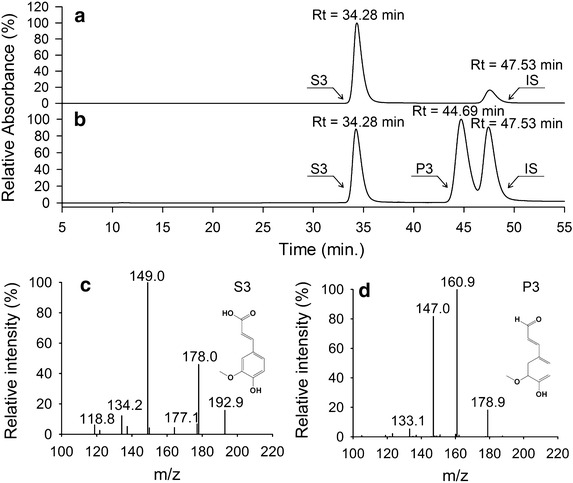
Production of coniferaldehyde in *E. coli* M-4CL1–CCR. **a** ferulic acid (S3), Internal standard (IS); **b** reaction product of ferulic acid (P3); **c** MS/MS profile of S3; **d** MS/MS profile of P3.

The metabolites produced during the biotransformation process were further analyzed using HPLC–PDA–ESI–MSn. As shown in Fig. 8, a new peak appeared (P1). The molecular mass of P1 was 148 Da, 16 Da less than the predicted molecular mass of S1, which corresponded with the reduction of a carboxyl group into an aldehyde group. This indicates that the *p*-coumaric acid was converted into *p*-coumaraldehyde by enzyme-catalyzed reduction. Biotransformation of caffeic acid by *E. coli* strains M-4CL1–CCR resulted in a new product (P2) with a HPLC retention time and molecular mass (164 Da) identical to caffealdehyde (Fig. 9b, d). As expected, the bifunctional enzyme converted caffeic acid into caffealdehyde. Similarly, the engineered strains produced coniferaldehyde (P3) when ferulic acid was supplemented in the medium. Figure 10 shows a new peak (Rt = 44.69 min) that corresponds to coniferaldehyde by comparison with the standard solution. The MS/MS spectrum of the reaction product matched authentic coniferaldehyde. Previous studies showed that 4CL1 from *P. tomentosa* was unable to catalyze the conversion of sinapic acid to sinapyl-CoA [20]. Therefore, all phenylpropanoic acids except for sinapic acid were converted into 4-hydroxycinnamaldehydes via the biosynthetic pathway.

### Quantitative analysis of 4-hydroxycinnamaldehydes

To quantify the productivity of this new metabolic system, culture samples were taken periodically, and the concentration of metabolites was measured. As shown in Fig. 6b, *p*-coumaric acid was the most effective acyl donor, producing approximately 49 mg/L of *p*-coumaraldehyde at 4 h. The decrease in *p*-coumaric acid during biotransformation is associated with the formation of *p*-coumaraldehyde. The maximum amount of extracellular caffealdehyde was produced at 8 h, approximately 19 mg/L (Fig. 6c). The accumulation of coniferaldehyde continued to increase until 32 h, while the ferulic acid content decreased. Approximately 35 mg/L of coniferaldehyde was produced at 32 h (Fig. 6d). The reduction in *p*-coumaraldehyde and caffealdehyde concentration over time was due to the loss of the active aldehydes to the headspace, which means that these reported production values are underestimated [19]. Thus it is necessary to halt the bioconversion reaction by a physical method after the product is no longer increased. Furthermore, it seems unlikely that these substrates are toxic to *E. coli* at the concentrations produced here. Above OD_600_ = 1, almost all of the 4-hydroxycinnamic acid was completely consumed after 32 h. From these data, we can calculate the conversion ratio and yield (Table 3). Our results indicate that the recombinant enzyme in this pathway was sufficiently active to catalyze this conversion.

**Table 3:** The highest yield of 4-hydrocinnaldehydes and the corresponding conversion ratio of phenylpropanoic acids

Substrate	Time (h)	Conversion ratio (%)	Product	Yield (%)
*p*-Coumaric acid	4	46	*p*-Coumaraldehyde	34
Caffeic acid	8	44	Caffealdehyde	11
Ferulic acid	32	89	Coniferaldehyde	19

## Discussion

4-Hydroxycinnamaldehydes are a class of key intermediates in plant secondary metabolism pathways, which are subsequently converted into hydroxycinnamyl alcohols (or monolignols) via a one-step reduction reaction. These monolignols are incorporated into the lignin polymer, which is essential for water transport, mechanical support and for plant defenses against pathogens [22, 23]. Cinnamicaldehyde is also one of the important spices commonly used in the modulation of gardenia, jasmine, rose and other essences. Moreover, these aldehydes are significant and valuable in medicinal science; for example, *p*-hydroxycinnaldehyde from *Alpinia galanga* is a potential therapeutic agent for treatment of osteoarthritis [24]. Extraction of nature products from plants requires prolonged plant cell growth, tedious separation procedures and considerable plant biomass. Traditional chemical synthetic methods involve multiple steps, which often result in a very low total yield [25]. More importantly, the synthesized products are sometimes of vastly inferior quality, often containing by-products. Biotransformation is an emerging technology in the field of plant natural product synthesis, due to its mild reaction conditions and environmentally friendly processes [26]. The major advantages of using whole-cells as metabolic engineering tools are the simple nutritional requirements of *E. coli* strains which are also easily cultured and the natural protective layer provided by these cell bodies which can effectively prevent enzyme from inactivation. Already there are reports of valuable compounds produced by recombinant *E. coli*; these include the antibiotic erythromycin, biodiesel and biodegradable plastic [27–30].

Biocatalysis can be performed using both isolated enzymes (in vitro) and whole-cells (in vivo). For in vitro systems, the enzymatic processes requiring an input of energy and the assistance of cofactors such as ATP, CoA and NADPH would be prohibitively expensive if used in equimolar amounts. The price of cofactors is currently a substantial barrier to industrialization. For in vivo systems, the enzymes involved in product formation and cofactor regeneration are often overexpressed in cells [31]. The success of recent work utilizing biocatalysts generated by microbes to produce 4-hydroxycinnamaldehydes has validated this point. Moreover, enzyme isolation and purification are time consuming and require more attention from researchers because in general it is difficult to carry out reactions requiring more than one enzyme, contrary to the use of whole cells [32].

We have demonstrated the feasibility of engineering *E. coli* to produce 4-hydroxycinnamaldehydes. The products from reduction of phenylpropanoic acids in vivo were pure, as evidenced by HPLC spectra (Figs. 8b, 9b, 10b). No by-product formation occurred in the whole bioconversion process, and the products can be purified economically. Multiple changes were made to *E. coli* for relevant cinnamaldehyde production, including overexpression of the genes encoding 4-coumaric acid: CoA ligase (4CL1) and cinnamoyl CoA (CCR) reductase from *P. tomentosa*. To our knowledge, this is the first biotransformation system using a fusion enzyme for 4-hydroxycinnamaldehyde production in *E. coli*.

Recombinant DNA technologies for creating and overexpressing a fusion gene can be used to improve the productivity of enzyme technology [33–35]. It is not feasible to produce 4-hydroxycinnamaldehydes using a mixture of individual native enzymes 4CL1 and CCR, because the intermediate product 4-hydroxycinnamoyl-CoA is unable to freely pass through the cellular membrane (Fig. 5a). Due to the natural barrier function of the cellular membrane, accessibility of substrates to intracellular enzymes and the export of products are limited, hampering sufficient whole-cell bioconversion [36]. The fusion enzyme 4CL1–CCR can catalyze sequential reactions, thereby avoiding the permeability problem of intermediates. In this regard, the fusion enzyme demonstrates its superiority over a mixture of individual native enzymes catalyzing the same multi-step sequential reaction. On the other hand, the fusion expression system showed in vivo enzymatic activity significantly higher than the co-expression system (Fig. 5b), presumably due to the close proximity of catalytic domains in the fusion protein that can improve the binding and the catalytic efficiency for the intermediate product (*p*-coumaroyl-CoA). When *p*-coumaroyl-CoAs were synthesized by 4CL1 and then gathered around the catalytic domains of CCR, their effective concentration in the active site was increased, and this therefore resulted in enhancing the catalytic efficiency of the fusion enzyme.

The catalytic efficiency of enzymes from different sources can vary considerably, especially for different substrates. The substrate preference of the fusion enzyme 4CL1–CCR leads to divergent reaction trends in an in vivo system (Fig. 6). Previous studies reported that 4CL1 from *P. tomentosa* exhibits the greatest catalytic efficiency with *p*-coumaric acid, followed by caffeic acid and ferulic acid, in in vitro systems. However, 4CL1 was unable to catalyze the conversion of sinapic acid to sinapyl-CoA for spatial and structural reasons [37]. To date, no substrate preference of CCR from *P. tomentosa* has been reported. By investigating the enzyme characteristics of CCR from *Medicago truncatula* and *Aspen*, researchers have found that CCR can utilize various hydroxycinnamoyl-CoA thioester substrates, although most of the characterized CCR isoforms exhibit a preference for feruloyl-CoA [38, 39]. The fusion enzyme 4CL1–CCR retains the enzymatic activity of its individual native enzymes, but its catalytic efficiency is variable. Under the combined influence of 4CL1 and CCR, *p*-coumaric acid achieved the highest and most rapid effect during biotransformation, followed by caffeic acid, with ferulic acid being the slowest. Taken together with the reaction rate and yield, *p*-coumaric acid is the most favorable substrate for this fusion enzyme in an in vivo system (Table 3).

The production of *p*-coumaraldehyde in a recombinant *E. coli* strain expressing 4CL1 and CCR upon introduction of IPTG was markedly higher than production in its absence (Fig. 4). This result indicates that overexpression of 4CL1–CCR leads to increased conversion of *p*-coumaric acid to *p*-coumaraldehyde. Therefore, we overexpressed 4CL1–CCR by adding IPTG to produce 4-hydroxycinnamaldehydes. However, there was still room for improvement in the capacity of the recombinant strain to synthesize aldehydes. Thereby, seeking effective measures to enhance the output, such as optimization of a two-phase (growth phase followed by production phase) bioprocess for balancing the enzyme expression, enzyme activity, cell growth and product formation should be further considered.

Hydroxycinnamyl alcohols can be synthesized from cinnamaldehydes by the action of cinnamyl alcohol dehydrogenase (CAD) [40]. Therefore, synthesis of *p*-coumaryl alcohol or coniferyl alcohol from *p*-coumarate acid or ferulic acid might be possible if a gene econding CAD was expressed in *E. coli* harboring 4CL1–CCR. Coniferyl alcohol is commercially available now, but is eight to tenfold more expensive than 4-hydroxycinnamaldehyde [41]. Introduction of an artificial metabolic pathway into *E. coli* would undoubtedly be an attractive method of alcohol production. However, the precursors are not renewable resources because phenylpropanoic acids are not synthesized in the wild type *E. coli* strain. A recent study reporting artificial biosynthesis of phenylpropanoic acids in a tyrosine-overproducing *E. coli* strain may provide a solution to this problem [42]. Thus, the bioconversion of hydroxycinnamyl alcohols from tyrosine may become possible in the near future.

In addition, we describe a highly sensitive and selective method for separation and identification of phenylpropanoic acids and their corresponding cinnamaldehydes in the present paper. The method includes crude extraction with ethyl acetate, pre-purification with a SPE cartridge, separation by HPLC and detection using an ESI-Ion trap-MS system. The feasibility of this method has been proven in the application of the method to the analysis of the metabolites of whole-cell catalysts. By applying this novel method, five existing aromatic compounds and two biosynthetic aldehydes were detected (Fig. 7). This method possesses the features of effective separation, sensitive detection and high reproducibility.

## Conclusions

In summary, we have demonstrated the in vivo bioconversion of phenylpropanoic acids to 4-hydroxycinnamaldehydes using *E. coli* overexpressing the bifunctional enzyme 4CL1–CCR. Overexpression of the fused recombinant proteins formed an artificial metabolic pathway in *E. coli*, thereby avoiding the membrane penetration challenge of intermediates. Using this strategy, 49 mg/L of *p*-coumaraldehyde, 19 mg/L of caffealdehyde and 35 mg/L of coniferaldehyde were produced. To improve the efficiency and applicability of this metabolic system, further efforts have to be directed at identifying the bottlenecks to maximize the flux and optimize the production conditions. To our knowledge, there are no reports in which the utilization of a double 4CL1, CCR derivative has been used to produce 4-hydroxycinnamaldehydes. This work utilizes recombinant whole-cell *E. coli* as the catalyst, which provides a novel route for the synthesis of 4-hydroxycinnamaldehydes. Furthermore, it is an example of biological synthesis of valuable plant natural products using engineered *E. coli*. Similar biological synthesis approaches can be applied for other plant metabolites.

## Methods

### Strains and reagents

The *E. coli* strains and plasmids used in this study are listed in Table 4. *E. coli* M15 (pREP4) and BL21 (DE3) cells were used for recombinant protein production. *E. coli* DH5α cells were used for plasmid cloning. All restriction enzymes and T4 DNA ligase were purchased from Takara (Shiga, Japan). Polymerase chain reaction (PCR) amplification was performed using Hotstart Taq DNA polymerase (Qiagen, Germany).

**Table 4:** Plasmids, *Escherichia coli* strains and primers used in this study

Plasmids or *E. coli* strains or primers	Relevant properties or genetic marker	Source
Plasmids		
pMD18-T	ColE1 ori, Amp^r^	Takara
pQE31	ColE1 ori, T5 promoter, Amp^r^	Qiagen
pETDuet-1	f1 ori, T7 promoter, Amp^r^	Novagen
pQ-4CL1	pQE31 carrying *4CL1* from *P. tomentosa*	This study
pQ-CCR	pQE31 carrying *CCR* from *P. tomentosa*	This study
pQ-4CL1–CCR	pQE31 carrying fusion gene *4CL1*–*CCR* from *P. tomentosa*	This study
pE-4CL1+CCR	pETDuet-1 carrying *4CL1* and *CCR* from *P. tomentosa*	This study
pE-4CL1–CCR	pETDuet-1 carrying fusion gene *4CL1*–*CCR* from *P. tomentosa*	This study
Strains		
DH5α	Cloning host	Qiagen
M15 (pREP4)	Expression host	Qiagen
BL21(DE3)	Expression host	Novagen
M-4CL1	M15 carrying pQ-4CL1	This study
M-CCR	M15 carrying pQ-CCR	This study
M-4CL1–CCR	M15 carrying pQ-4CL1–CCR	This study
B-4CL1+CCR	BL21(DE3) carrying pE-4CL1+CCR	This study
B-4CL1–CCR	BL21(DE3) carrying pE-4CL1–CCR	This study
Primers		
pQ-4CL1-F	cgcaatggacgccacaatgaat	
pQ-4CL1-R	actgtcttacgttgggtacg	
pQ-CCR-F	atgccggttgatgcttcatc	
pQ-CCR-R	gaggccttattgaatcttc	
pQ-4CL1–CCR-F	ggcatgcatgaatccacaagaagaattc (*Sph*I site is underlined)	
pQ-4CL1–CCR-R	gatgaagcatcaaccggcatagaaccaccaccaccagaaccaccaccaccagaaccaccaccacctatgcctgccaactttt*ctttcag*	
pQ-4CL1–CCR-F	atgccggttgatgcttcatcac*tttcag*	
pQ-4CL1–CCR-R	gggtaccttattgaatcttcacagactcttc (*Kpn*I site is underlined)	
pE-4CL1-F	ttggcgcgccatgcatgaatccacaagaagaa (*Asc*I site is underlined)	
pE-4CL1-R	cttaagaattatgcctgccaacttttctttc (*Afl*II site is underlined)	
pE-CCR-F	ggggtaccatgccggttgatgcttcatcacttt (*Kpn*I site is underlined)	
pE-CCR-R	ccttaattaattattgaatcttcacagactcttct (*Pac*I site is underlined)	
pE-4CL1–CCR-F	ttggcgcgccatgcatgaatccacaagaagaa (*Asc*I site is underlined)	
pE-4CL1–CCR-R	cttaagaattattgaatcttcacagactcttct (*Afl*II site is underlined)	

*F* forward primer, *R* reverse primer.

### Construction of recombinant expression vector and cultivation conditions of *E. coli*

The genes for 4CL1 and CCR were cloned from *P. tomentosa* and kept in the laboratory. PCR was carried out using primers designed on the basis of the full-length cDNA sequences of 4CL1 and CCR. The primer sequences for the *4CL1*, *CCR* and fusion gene *4CL1*–*CCR* are shown in Table 4. The cDNA sequences of 4CL1 and CCR were subcloned into pQE31 plasmid. Each recombinant plasmid (pQ-4CL1 and pQ-CCR; see Table 4) was transformed into *E. coli* M15 cells, and co-cultured in Luria–Bertani (LB) medium containing 100 μg/mL ampicillin and 25 μg/mL kanamycin and grown at 37 °C.

For the construction of 4CL1–CCR fusion gene expression vector, we designed Primer pQ-4CL1–CCR-F^1^ and pQ-4CL1–CCR-R^1^, into which a *Sph*I site (under line) and overlapping region (italic font) was respectively introduced and primer pQ-4CL1–CCR-F^2^ and pQ-4CL1–CCR-R^2^, into which overlapping region (italic font) and a *Kpn*I site (under line) was introduced. The linker sequence (wavy line) was introduced into primer pQ-4CL1–CCR-R^1^ to construct the fusion gene of bifunctional enzyme. The multiple PCR products were recycled and connected with a pMD18-T vector to construct a recombinant plasmid pMD18-T-4CL1–CCR. After confirmed by DNA sequencing, the amplified products were digested with *Sph*I (underlined) and *Kpn*I (underlined) and then cloned into the same sites of a pQE31 expression vector. The fusion gene expression vector was transformed into *E. coli* M15 cells, and cultured in LB medium containing 100 μg/mL ampicillin and 25 μg/mL kanamycin and grown at 37 °C.

For the co-expression vector pE-4CL1+CCR construct, the gene sequence encoding 4CL1 was amplified from pQ-4CL1 and subcloned into the *Asc*I/*Afl*II sites of pETDuet-1 vector, and then the gene sequence encoding CCR was amplified from pQ-CCR and subcloned into a second cloning site, the *Kpn*I/*Pac*I site of pETDuet-1. The resulting recombinant plasmid was confirmed by sequencing. The co-expression vector was transformed into *E. coli* BL21 (DE3) cells, and cultured in LB medium containing 100 μg/mL ampicillin and grown at 37 °C.

The fusion gene *4CL1*–*CCR*, which was cloned into pQE31 plasmid previously, was amplified using PCR, and subcloned into the *Asc* I/*Afl* II sites of pETDuet-1. The resulting recombinant plasmid pE-4CL1–CCR was transformed into *E. coli* BL21 (DE3) cells, and cultured in LB medium containing 100 μg/mL ampicillin and grown at 37 °C.

Cell growth was monitored by measuring the OD at 600 nm (Biomate 3S UV–Visible Spectrophotometer, Thermo scientific). Protein expression was induced with 0.4 mM isopropyl-β-d-thiogalactoside (IPTG) when cell optical density (OD_600_) reached 0.6. The incubation was continued for another 8 h at 28 °C. *E. coli* concentrations were monitored throughout the culture.

### SDS-PAGE analysis of recombinant enzymes

The cells were then harvested by centrifugation at 5,000 rpm for 15 min. The total crude proteins extracted from different strains were separated by SDS-PAGE. The SDS-PAGE was carried out with the 7 cm slab gel apparatuses using 12 % polyacrylamide as the separating gel and 4 % polyacrylamide as the stacking gel. Protein samples were mixed with 10× loading buffer, boiled for 5 min, and then loaded per lane. The mixed samples were separated for 30 min at 80 V and then at 120 V until the bromophenol blue dye front reached the bottom of the gel. After electrophoresis, proteins were visualized by staining with Coomassie brilliant blue (R-250). Gel images were captured and analyzed with Quantity One software (Bio-Rad).

### Production of 4-hydroxycinnamaldehydes in vivo

Three kinds of phenylpropanoic acids (*p*-coumaric acid, caffeic acid and ferulic acid) were directly added into the culture medium, with a final concentration of 1 mM, respectively. There was no additional cofactor added during the biotransformation process. The cultures were grown in flasks shaked at 200 rpm, 37 °C for 32 h.

### Extraction and purification of metabolites

For the metabolites identification and quantification, 5 mL culture samples were centrifuged at 12,000 rpm for 3 min. Sinapaldehyde was added to supernatant as the internal standard. The supernatant containing the internal standard was extracted three times with an equal volume of ethyl acetate. The extracts were dried under a stream of nitrogen.

Extract samples were redissolved in 2 mL 5 % (V/V) methanol and then loaded onto an activated Oasis MCX SPE cartridge by gravity flow. The SPE cartridge were preactivated with 5 mL methanol and 5 mL acetic acid, and then washed with 5 mL 0.1 M acetic acid. The target metabolites were eluted with 5 mL of 0.1 M acetic acid in 40 % (V/V) acetonitrile. The purified metabolites were dried under vacuum, redissolved in 200 μL of 50 % (V/V) methanol, and ultral-filtered through a micro-filter (4 mm, 0.25 μm), before injected into HPLC–PDA–ESI–MSn analysis.

### Detection and identifically characterization of metabolites by HPLC–PDA–ESI–MSn

An HPLC system, consisting of Surveyor Autosampler, Surveyor LC pump, Surveyor Photo Diode Array (PDA) detector (Thermo Finnigan, Waltham, MA, USA), and a reversed-phase column (ZORBAX 300SB-C18, 2.1 × 150 mm, 3.5 μm; Agilent, Santa Clara, CA, USA) was used to separate the metabolites. 10 μL of samples were injected for the analysis throughout. The gradient profile was 8 % B for 2 min, increased to 20 % B in 38 min, then to 100 % B in 12 min and maintained for 10 min, and decreased to 8 % B in 2 min and maintained for 10 min (A = 0.1 % formic acid in water, B = 100 % acetonitrile). The flow rate was 0.15 mL/min. The acquisition time was 55 min and delay time was 5 min per spectrum. The separation was monitored at 325 nm.

An ion trap mass spectrometer (LCQ DECA XP MAX) coupled with an ESI source (Thermo Finnigan) was used to identify the metabolites. The MS parameters were the following: sheath gas (nitrogen) flow rate, 40 arb; aux/sweep gas (nitrogen) flow rate, 10 arb; spray voltage, 4.5 kV; capillary temperature, 320 °C. Collision energy and other tune parameters were optimized for dissociation of parent ions into product ions for each metabolite. The mass spectrometer was acquired in data-dependent MS/MS mode: each full MS scan (in the range 100–220 *m*/*z*) was followed by three MS/MS of selected ions.

### Quantification of 4-hydroxycinnamaldehydes

Data processing was performed in Xcalibur 2.1 (Thermo Finnigan). The quantitative determination of substrate and product in the transformation system were performed with external standard and internal standard method. The systematic errors throughout the entire experiment were corrected according to internal standard, including sample extraction and purification, chromatography and MS detection. Pure samples of various metabolites with concentrations ranging from 3.0 to 15,000.0 pmol were used as standards for calibration. Calibration curves were calculated by plotting the peak area. The metabolites were quantified via seven-point calibration curves of authentic standard compounds for which the *R*^2^ coefficients were ≥0.99. The regression equation and linearity range were presented in Table 1. Each analysis was performed in triplicate. Because *p*-coumaraldehyde and caffealdehyde are not commercially available, we used sinapaldehyde to generate a standard curve for quantitative analysis of the reaction products.

## Abbreviations

4CL1: 4-coumaric acid: coenzyme A ligase
CCR: cinnamoyl coenzyme A reductase
HPLC–PDA–ESI–MSn: high performance liquid chromatography-photo diode array-electrospray ionization-multistage mass spectrometry
ATP: adenosine triphosphate
CoA: coenzyme A
NADPH: reduced form of nicotinamide adenine dinucleotide phosphate
PCR: polymerase chain reaction
LB: Luria–Bertani
IPTG: isopropyl-β-d-thiogalactoside
SDS-PAGE: sodium dodecyl sulphate polyacrylamide gel electrophoresis
MS/MS: tandem mass spectrometry

## References

[1] Vanholme R, Demedts B, Morreel K, Ralph J, Boerjan W (2010). Lignin biosynthesis and structure. Plant Physiol.

[2] Kim H, Ralph J, Lu F, Ralph SA, Boudet AM, MacKay JJ (2003). NMR analysis of lignins in CAD-deficient plants. Part 1. Incorporation of hydroxycinnamaldehydes and hydroxybenzaldehydes into lignins. Org Biomol Chem.

[3] Lynn DG, Chang M (1990). Phenolic signals in cohabitation: implications for plant development. Annu Rev Plant Physiol Plant Mol Biol.

[4] Banjerdpongchai R, Punyati P, Nakrob A, Pompimon W, Kongtawelert P (2011). 4′-Hydroxycinnamaldehyde from *Alpinia galanga* (Linn.) induces human leukemic cell apoptosis via mitochondrial and endoplasmic reticulum stress pathways. Asian Pac J Cancer Prev.

[5] Iliefski T, Li S, Lundquist K (1998). Synthesis of cinnamaldehydes by oxidation of arylpropenes with 2,3-dichloro-5,6-dicyanoquinone. Acta Chem Scand.

[6] Li LG, Jacqueline LP, Toshiaki U, Vincent C (2000). 5-Hydroxyconiferyl aldehyde modulates enzymatic methylation for syringyl monolignol formation, a new view of monolignol biosynthesis in *Angiosperms*. J Biol Chem.

[7] Quideau S, Ralph J (1992). Facile large-scale synthesis of coniferyl, sinapyl, and *p*-coumaryl alcohol. J Agric Food Chem.

[8] Zhu Y, Mohammadi A, Ralph J (2011). Facile synthesis of 4-hydroxycinnamaldehydes. Bioenergy Res.

[9] Olstein R, Stephenson EFM (1979). The synthesis of methoxy- and hydroxy-substituted cinnamaldehydes and their corresponding epoxides. Cinnamaldehydes.

[10] Fowler ZL, Gikandi WW, Koffas MAG (2009). Increased malonyl coenzyme A biosynthesis by tuning the *Escherichia coli* metabolic network and its application to flavanone production. Appl Environ Microbiol.

[11] Huang D, Wen JP, Wang GY, Yu GH, Jia XQ, Chen Y (2012). In silico aided metabolic engineering of *Streptomyces roseosporus* for daptomycin yield improvement. Appl Microbiol Biotechnol.

[12] Pickens LB, Tang Y, Chooi YH (2011). Metabolic engineering for the production of natural products. Ann Rev Chem Biomol Eng.

[13] Keasling JD (2010). Manufacturing molecules through metabolic engineering. Science.

[14] Horinouchi S (2008). Combinatorial biosynthesis of nonbacterial and unnatural flavonoids stilbenoids and curcuminoids by microorganisms. J Antibiot.

[15] Fowler ZL, Koffas MAG (2009). Biosynthesis and biotechnological production of flavanones: current state and perspectives. Appl Microbiol Biotechnol.

[16] Zhang H, Stephanopoulos G (2012). Engineering *E. coli* for caffeic acid biosynthesis from renewable sugars. Appl Microbiol Biotechnol.

[17] Kim B, Jung WD, Mok H, Ahn J (2013). Production of hydroxycinnamoyl-shikimates and chlorogenic acid in *Escherichia coli*: production of hydroxycinnamic acid conjugates. Microb Cell Fact.

[18] Eudes A, Juminaga D, Baidoo EE, Collins FW, Keasling JD, Loque D (2013). Production of hydroxycinnamoyl anthranilates from glucose in *Escherichia coli*. Microb Cell Fact.

[19] Martin VJJ, Pitera DJ, Withers ST, Newman JD, Keasling JD (2003). Engineering a mevalonate pathway in *Escherichia coli* for production of terpenoids. Nat Biotechnol.

[20] Fan BY, Lu H, Jiang XN (2007). High-level expression of 4-coumarate:coenzyme A ligase gene Pt4CL1 of Populus tomentosa in E. coli. For Stud China.

[21] Wang DD, Bai H, Chen WQ, Lu H, Jiang XN (2009). Identifying a cinnamoyl coenzyme A reductase (CCR) activity with 4-coumaric acid: coenzyme a ligase (4CL) reaction products in *Populus tomentosa*. J Plant Biol.

[22] Zhao Q, Dixon RA (2011). Transcriptional networks for lignin biosynthesis: more complex than we thought?. Trends Plant Sci.

[23] Shi R, Sun YH, Li Q, Heber S, Sederoff R, Chiang VL (2009). Towards a systems approach for lignin biosynthesis in *Populus trichocarpa* transcript abundance and specific city of the monolignol biosynthetic genes. Plant Cell Physiol.

[24] Phitak T, Choocheep K, Pothacharoen P, Pompimon W, Premanode B, Kongtawelert P (2009). The effects of *p*-hydroxycinnamaldehyde from *Alpinia galanga* extracts on human chondrocytes. Phytochemistry.

[25] Chen F, Kota P, Blount JW, Dixon RA (2001). Chemical syntheses of caffeoyl and 5-OH coniferyl aldehydes and alcohols and determination of lignin *O*-methyltransferase activities in dicot and monocot species. Phytochemistry.

[26] Schmid A, Dordick J, Hauer B, Kiener A, Wubbolts M, Witholt B (2001). Industrial biocatalysis today and tomorrow. Nature.

[27] Pfeifer BA, Admiraal SJ, Gramajo H, Cane DE, Khosla C (2001). Biosynthesis of complex polyketides in a metabolically engineered strain of *E. coli*. Science.

[28] Kalscheuer R, Stölting T, Steinbüchel A (2006). Microdiesel: *Escherichia coli* engineered for fuel production. Microbiology.

[29] Van Wegen RJ, Lee SY, Middelberg APJ (2001). Metabolic and kinetic analysis of poly(3-hydroxybutyrate) production by recombinant *Escherichia coli*. Biotechnol Bioeng.

[30] Bokinsky G, Peralta-Yahya PP, George A, Holmes BM, Steen EJ, Dietrich J (2011). Synthesis of three advanced biofuels from ionic liquid-pretreated switchgrass using engineered *Escherichia coli*. Proc Natl Acad Sci USA.

[31] Chemler JA, Fowler ZL, McHugh KP, Koffas MAG (2010). Improving NADPH availability for natural product biosynthesis in *Escherichia coli* by metabolic engineering. Metab Eng.

[32] De Carvalho CCCR (2011). Enzymatic and whole cell catalysis: finding new strategies for old processes. Biotechnol Adv.

[33] Seo HS, Koo YJ, Lim JY, Song JT, Kim CH, Kim JK (2000). Characterization of a bifunctional enzyme fusion of trehalose-6-phosphate synthetase and trehalose-6-phosphate phosphatase of *Escherichia coli*. Appl Environ Microbiol.

[34] Corson TW, Aberle N, Crews CM (2008). Design and applications of bifunctional small molecules-Why two heads are better than one. ACS Chem Biol.

[35] Wang Y, Yi H, Wang M, Yu O, Jez JM (2011). Structural and kinetic analysis of the unnatural fusion protein 4-coumaroyl-CoA ligase::stilbene synthase. J Am Chem Soc.

[36] Chen RR (2007). Permeability issues in whole-cell bioprocesses and cellular membrane engineering. Appl Microbiol Biotechnol.

[37] Hu Y, Gai Y, Yin L, Wang X, Feng C, Feng L (2010). Crystal structures of a *Populus tomentosa* 4-coumarate:CoA ligase shed light on its enzymatic mechanisms. Plant Cell.

[38] Zhou R, Jackson L, Shadle G, Nakashima J, Temple S, Chen F (2010). Distinct cinnamoyl CoA reductases involved in parallel routes to lignin in *Medicago truncatula*. Proc Natl Acad Sci.

[39] Li LG, Cheng XF, Lu SF, Tomoyuki N, Toshiaki U, Vincent C (2005). Clarification of cinnamoyl co-enzyme A reductase catalysis in monolignol biosynthesis of *Aspen*. Plant Cell Physiol.

[40] Chao N, Liu SX, Liu BM, Li N, Jiang XN, Gai Y (2014). Molecular cloning and functional analysis of nine cinnamylalcohol dehydrogenase family members in *Populus tomentosa*. Planta.

[41] Kim H, Ralph J (2005). Simplified preparation of coniferyl and sinapyl alcohols. J Agric Food Chem.

[42] Kang SY, Choi O, Lee JK, Hwang BY, Uhm TB, Hong YS (2012). Artificial biosynthesis of phenylpropanoic acids in a tyrosine overproducing *Escherichia coli* strain. Microb Cell Fact.

